# The value of plasma metanephrine measurements during adrenal vein sampling

**DOI:** 10.1530/EC-23-0300

**Published:** 2024-01-12

**Authors:** Richard W Carroll, Brian Corley, Joe Feltham, Patricia Whitfield, William Park, Rowena Howard, Melissa Yssel, Ian Phillips, Simon Harper, Jun Yang

**Affiliations:** 1 Endocrine, Diabetes, and Research Centre, Wellington Regional Hospital, New Zealand; 2 Department of Medicine, University of Otago, Wellington, New Zealand; 3 Department of Radiology, Wellington Regional Hospital, New Zealand; 4 University of Otago, Wellington, New Zealand; 5 Diabetes and Endocrinology Service, Hutt Hospital, New Zealand; 6 Department of Biochemistry & Endocrinology, Awanui Labs, New Zealand; 7 Department of Biochemistry, Awanui Labs, Dunedin, New Zealand; 8 Department of Surgery & Anaesethesia, University of Otago, Wellington, New Zealand; 9 Department of General Surgery, Wellington Regional Hospital, New Zealand; 10 Centre for Endocrinology and Metabolism, Hudson Institute of Medical Research, Clayton, Victoria, Australia; 11 Department of Medicine, Monash University, Clayton, Victoria, Australia

**Keywords:** adrenal vein sampling, primary aldosteronism, metanephrines

## Abstract

**Objective:**

The assessment of primary aldosteronism incorporates adrenal vein sampling (AVS) to lateralize aldosterone excess. Current adrenal vein sampling protocols rely on concurrent cortisol measurements to assess successful cannulation and lateralization and may be inaccurate in the setting of autonomous cortisol secretion. We aimed to compare the measurement of plasma cortisol and metanephrine concentrations to assess cannulation and lateralization during AVS.

**Design:**

This is a diagnostic accuracy study in a tertiary referral endocrinology department.

**Methods:**

Forty-one consecutive patients with confirmed primary aldosteronism undergoing AVS (49 procedures) were included. None had cortisol autonomy. The use of plasma metanephrine-based ratios were compared with standard cortisol-based ratios to assess cannulation and lateralization during ACTH-stimulated AVS.

**Results:**

There was strong agreement between a cortisol selectivity index (SI) ≥5.0 and an adrenal vein (AV) to peripheral vein (PV) plasma metanephrine ratio (AVmet–PVmet) of ≥12.0 to indicate successful cannulation of the AV (*n* = 117, sensitivity 98%, specificity 89%, positive predictive value (PPV) 95%, negative predictive value (NPV) 94%). There was strong agreement between the standard cortisol-based SI and an AV plasma metanephrine-to-normetanephrine ratio (AVmet–AVnormet) of ≥2.0 to indicate successful cannulation (*n* = 117, sensitivity 93%, specificity 86%, PPV 94%, NPV 84%). There was strong agreement between the cortisol- or metanephrine-derived lateralization index (LI) > 4.0 for determining lateralization (*n* = 26, sensitivity 100%, specificity 94.1%, PPV 91.6%, NPV 100%).

**Conclusions:**

Ratios incorporating plasma metanephrines provide comparable outcomes to standard cortisol-based measurements for interpretation of AVS. Further studies are required to assess the use of metanephrine-derived ratios in the context of confirmed cortisol autonomy.

**Significance statement:**

Primary aldosteronism is a common cause of secondary hypertension, and adrenal vein sampling remains the gold standard test to assess lateralization. Cortisol-derived ratios to assess cannulation and lateralization may be affected by concurrent cortisol dysfunction, which is not uncommon in the context of primary aldosteronism. Our study showed comparable outcomes when using accepted cortisol-derived or metanephrine-derived ratios to determine cannulation and lateralization during adrenal vein sampling. Further research is required to validate these findings and to assess the use of metanephrine-derived ratios in the context of confirmed concurrent cortisol dysfunction.

## Introduction

Primary aldosteronism (PA) is associated with hypertension and hypokalemia and an increased morbidity in comparison to essential hypertension (1, 2, 3). The prevalence of PA is reported to be at least 6% in the adult hypertensive population, and up to 30% in those assessed at referral centers (4, 5, 6, 7, 8). Once primary aldosteronism is biochemically confirmed, investigations are undertaken to differentiate between unilateral and bilateral disease, with the former potentially amenable to surgical resection (1). Surgery usually normalizes hypokalemia if present and significantly improves or resolves associated hypertension (9, 10). Thus, primary aldosteronism is the most common potentially curable form of hypertension (11).

Adrenal vein sampling (AVS) remains the gold standard investigation to lateralize hyperaldosteronism and therefore confirm whether the hyperaldosteronism relates to unilateral or bilateral adrenal gland disease (1, 12). Due to the high prevalence of nonfunctioning adrenal incidentalomas, many centers recommend AVS in all patients with confirmed PA who wish to pursue surgery, while others recommend AVS in all patients with confirmed PA above an age threshold, and only in younger patients if imaging does not clearly indicate lateralizing adrenal pathology (1, 13, 14, 15, 16, 17). Current AVS protocols incorporate measurement of cortisol concentrations from the adrenal veins (AV) and peripheral veins (PV), to assess for adequate cannulation of each adrenal vein, and lateralization of aldosterone excess (1, 16, 17). Synacthen may be infused throughout the procedure to increase cortisol output (16, 17). A synacthen stimulated AV:PV cortisol ratio (cortisol-derived selectivity index, SIcort) of ≥3-5 has been validated in several studies to indicate adequate cannulation (1, 16, 17). Once adequate cannulation is confirmed, an aldosterone-to-cortisol ratio in one AV that is four times higher than the contralateral aldosterone-to-cortisol ratio (cortisol-derived lateralization index, LIcort ≥4) is considered sufficient lateralization to indicate unilateral disease (1, 16, 17).

However, the use of cortisol measurements in this context assumes normal cortisol physiology. Any clinical scenario where this is not the case could potentially lead to erroneous conclusions being drawn from the AVS data. Current or historical use of glucocorticoid therapy or intrinsic pituitary or adrenal dysfunction may lead to either a relatively lower corticosteroid concentration in the AV, lowered cortisol levels in general, or a blunted response to synacthen stimulation (18). Of particular relevance to patients with primary aldosteronism, the prevalence of cortisol co-secretion is high at between 13 and 33% (19, 20, 21, 22, 23). Tests to exclude concurrent cortisol autonomy are therefore recommended in advance of AVS, particularly in the context of larger nodules (>15 mm) where cortisol autonomy is more frequently reported (24). However, the optimal method for assessing for possible cortisol dysfunction in this context is yet to be established, and current testing strategies are limited (25, 26).

Previous studies have therefore assessed whether the use of plasma metanephrine measurements during AVS procedures is comparable to the use of cortisol in determining adequate cannulation. Norepinephrine is synthesized predominantly in extra-adrenal neurotransmitter tissue through dopamine-β-monooxygenase action on dopamine (27). Norepinephrine is converted by methylation to epinephrine, a reaction catalyzed by the enzyme phenylethanolamine *N*-methyltransferase (PNMT). As this enzyme is expressed primarily in the adrenal medulla, almost all epinephrine is of adrenal gland origin, although small amounts are synthesized in the brain and cardiac tissue (27). Norepinephrine and epinephrine are degraded to normetanephrine and metanephrine, respectively, by monoamine oxidase (MOA) and catechol-*O*-methyl transferase (COMT), which are both expressed widely, including within the chromaffin cells of the adrenal medulla (27). Dekkers and colleagues have shown that an AV to PV plasma metanephrine ratio (AVmet–PVmet) of ≥12 is comparable to standard cortisol based measurements to assess adequate cannulation (28). Goupil and colleagues published on the helpful application of this approach in the context of a patient with confirmed co-secretion of aldosterone and cortisol (29). Buffalo and colleagues have recently demonstrated that 18% of their cohort undergoing AVS had asymmetrical cortisol secretion, and further assessed the value of metanephrine measurements for both SI and LI assessments (30). Furthermore, the measurement of a free to total metanephrine concentration ratio appears to be noninferior to assessments of free metanephrine alone (31).

The present study therefore aimed to assess the application of previously described plasma metanephrine-derived selectivity indices (SImet) in PA subtyping, compared to the standard cortisol based approach. In addition, and given that epinephrine is predominantly of adrenal origin, we wished to assess whether the difference between AVmet and PVmet in a successfully cannulated vein was significantly greater than the difference between AVnormet and PVnormet. If so, we aimed to explore whether the use of an adrenal vein plasma metanephrine-to-normetanephrine ratio (AVmet–AVnormet), as an alternative method for assessing central vs peripheral concentrations, was comparable to the use of plasma metanephrine concentrations alone in assessing cannulation success. Finally, we compared the use of an aldosterone-to-metanephrine ratio (LImet) to the standard aldosterone-to-cortisol ratio (LIcort) to assess lateralization of aldosterone excess.

## Materials and methods

### Participants

All patients presenting consecutively for AVS at Wellington regional hospital between 2015 and 2021 were included. Patients provided informed consent for the saline infusion test and adrenal vein sampling procedure. As the care provided was standard clinical care, and we compared obtained results retrospectively against published results, ethical approval was not required for this study. The diagnosis of PA was established as per recommended practice guidelines and on the basis of local laboratory cutoffs (1). Patients with a positive screening aldosterone: renin ratio (aldosterone pmol/L–renin mIU/L ratio greater than or equal to 30.5 at the time of the study) underwent a seated saline infusion test under appropriate testing conditions (1). PA was confirmed if the aldosterone concentration was greater than or equal to 210 pmol/L at the end of the infusion. An aldosterone concentration less than 140 pmol/L was considered a normal response, while aldosterone concentrations between 140 and 210 pmol/L were considered indeterminate. If hypokalaemia was present, potassium was included in the saline infusion to prevent inaccurate results as per previous reports (32). Patients were selected for AVS once PA had been confirmed by the saline infusion test, and either (i) the patient was older than or equal to 40 years of age or (ii) less than 40 years of age without lateralizing pathology on imaging (1, 16, 17, 24). Concurrent cortisol autonomy was excluded prior to performing AVS through measurement of urinary free cortisol levels (normal range, 50–300 nmol/L/24 h), salivary cortisol levels (<10 nmol/L), or assessing the response to the 1 mg overnight dexamethasone suppression test (<50 nmol/L at 09:00 h). If investigating clinicians had no clinical suspicion for concurrent cortisol autonomy, or imaging was normal or showed an adenoma <15 mm, further investigations were sometimes not performed (24). A recent history of exogenous steroid exposure was excluded in all individuals.

### Adrenal vein sampling protocol

AVS was performed by an interventional radiologist as a day procedure, under local anesthesia and minor sedation, and after further informed consent had been obtained. Most antihypertensive agents were withheld for two weeks prior to the procedure, and mineralocorticoid antagonists for four weeks; alpha blockers and potassium replacement were continued if required. An ACTH infusion (50 µg/h) was commenced via a peripheral line 30 min prior to commencement of the procedure and continued until the procedure was terminated. Angled catheters were advanced up the inferior vena cava via a femoral vein puncture site, and the right and left Av then entered sequentially. Once in the veins, 3 × 3 mL blood samples were obtained, along with a 10 mL peripheral sample from the antecubital fossa. Samples were immediately sent to the laboratory for determination of cortisol, aldosterone, and metanephrine levels. Point-of-care cortisol measurements were not available to our radiologist during the study period.

### Interpretation of adrenal vein sampling data

Successful cannulation of each AV was defined as an AV–PV SIcort ≥5.0 (1, 16, 17, 24). If successful cannulation was confirmed, aldosterone excess was considered to be lateralized (LIcort) if the AV aldosterone-to-cortisol ratio on one side was ≥4 times the contralateral ratio (1, 16, 17, 24). For the purposes of this study, cannulation was assessed through the use of an adrenal vein-to-peripheral vein plasma metanephrine ratio (AVmet–PVmet) and an adrenal vein plasma metanephrine-to-normetanephrine ratio (AVmet–AVnormet), while lateralization was assessed through comparing the calculated AV aldosterone-to-metanephrine ratio from one adrenal vein to the contralateral vein (LImet). We compared our data against the AVmet–PVmet ratio proposed by Dekkers *et al.* in their earlier study (28). In the setting of multiple samples taken within a procedure and bilateral cannulation occurred for more than one sampling, the first successful sample was used to assess LImet.

### Biochemical analysis

EDTA plasma samples for aldosterone, renin, and plasma metanephrine/normetanephrine were centrifuged at room temperature, separated and frozen immediately, and transported deep-frozen to the reference laboratory. Plasma aldosterone concentration (PAC) was measured in EDTA plasma using the automated quantitative IDS-iSYS® Immunochemiluminometric (ICMA) platform (reportable range 102–3656 pmol/L (3.7–132 ng/dL) with a limit of detection (LoD) of 102.5 pmol/L and a limit of the blank (LoB) of 63.7 pmol/L, intra-assay coefficient of variation (%CVa) of 39.83% at 61.53 pmol/L), referenced to liquid chromatography–tandem mass spectrometry (LC-MS/MS). Plasma renin concentration (PRC) was measured in EDTA plasma using the quantitative IDS-iSYS® direct renin kit based on a two-sited (sandwich) chemiluminescence immunoassay (CLIA) (reportable range 1.8–550 mIU/L, with an analytical sensitivity of 0.6 mIU/L, LoD of 1.8, and limit of quantitation (LoQ) of 5.0 mIU/L; (%CVa) of 8.7% at 10.5 mIU/L), calibrated to the WHO International Standard 68/356.

Plasma metanephrines/normetanephrines were measured in EDTA plasma using an Agilent MS6490 Triple Quadrupole LC/MS system (Agilent Technologies®) with solid-phase extraction, RECIPE ClinMass® calibrators, and internal standards. Calibration ranges for normetanephrine are 5–10,666 pmol/L and 5–7093 pmol/L for metanephrines. Quality control target CVs (Canterbury Health Laboratories data) are low 628 pmol/L (CV 12.6%), mid 2079 pmol/L (CV 10.0%), and high 5169 pmol/L (CV 10.0%) for normetanephrine and low 295 pmol/L (CV 12.4%), mid 1207 pmol/L (CV 10.1%), and high 3879 pmol/L (CV 10.0%) for metanephrines. Current reference intervals for normetanephrine and metanephrines are <900 pmol/L and <500 pmol/L, respectively.

SST serum samples were collected for the measurement of cortisol and centrifuged at room temperature prior to analysis on the Roche Cobas e602 (competitive electrochemiluminescence immunoassay (ECLIA), measuring range 1.5–1750 nmol/L as defined by the limit of detection (LoD) and the maximum of the master curve).

### Statistical analysis

Variables are presented as means ± s.d. or median and interquartile range for non-normally distributed data. The sensitivity, specificity, positive predictive (PPV), and negative predictive values (NPV) were derived for each ratio based on published cutoffs to determine successful cannulation or lateralization which were then compared to traditional cortisol based ratios. All analyses were conducted in RStudio (https://www.r-project.org/). Receiver operator curves were created using the pROC and plotROC packages and used to determine the optimum cutoff value for each ratio.

## Results

### Patient and AVS characteristics

Forty-one patients with a total of 49 AVS procedures were included in the analysis. Patient characteristics and baseline data are presented in Table 1. Eight patients (20%) underwent a second procedure after unsuccessful cannulation during the first. Data were available for 120 attempted AV cannulations (50 left AV, 70 right AV), of which 83 were successful based on the SIcort (49(59%) left AV, 34 (49%) right AV). Plasma metanephrine and normetanephrine data were available from 117 out of 120 (98%) cannulations (not available from one left AV and two right AV) (see Table 2). Successful cannulation of both adrenal veins was achieved in 28 (68%) participants, of which 11 (39%) were subsequently diagnosed with unilateral and 17 (61%) with bilateral hyperaldosteronism. Thereafter, 10 out of 11 (91%) participants with unilateral hyperaldosteronism on AVS underwent adrenalectomy, all of whom achieved biochemical cure. The remaining patient with unilateral hyperaldosteronism moved elsewhere prior to surgery being offered and was lost to follow-up. Two patients proceeded to surgery despite unsuccessful AVS. One patient had a left adrenal adenoma on imaging and requested surgery given her young age (32), while the other (65, male) had a right adrenal adenoma on imaging but did not meet the threshold to confirm successful cannulation of the right adrenal vein during AVS (right vein SI = 1.4). Despite this, the LI strongly supported right-sided PA (55.8) and the patient requested surgery after discussion. Both patients achieved biochemical and clinical cure.

**Table 1:** Baseline characteristics of participants.

*n* = 41	Baseline
Age (years)	52.9 ± 12.5
Male (%)	26 (63)
Screening aldosterone (pmol/L)	559 (512)
Screening renin (mIU/L)	5.4 (6.65)
Screening ARR	85.8 (73.8)
T240 SIT aldosterone (pmol/L)	346 (325)
Lateralizing abnormality on CT imaging (%)	14 (34)
Assessment of cortisol autonomy (%)	36 (88)
Participants with confirmed concurrent cortisol autonomy (%)	0 (0)

Screening ARR = aldosterone (pmol/L)–renin (mIU/L) ratio (normal <30.5), T240 SIT aldosterone = aldosterone (pmol/L) at 240 min after the infusion of 2000 mL 0.9% saline. Biochemical assessments are presented as median (interquartile range).

**Table 2:** Biochemical samples obtained from successfully and unsuccessfully cannulated adrenal veins.

*n* = 117	Adrenal vein concentration (median (IQR))	Peripheral vein concentration (median (IQR))
**Successfully cannulated adrenal veins (*****n***** = 83)**		
Plasma aldosterone (pmol/L)	53,700 (19,400–92,075)	1040 (746–1655)
Serum cortisol (nmol/L)	14,515 (10,958–19,578)	653 (583–728)
Plasma normetanephrine (pmol/L)	3,729 (2492–6033)	428 (330–548)
Plasma metanephrine (pmol/L)	14,498 (8450–24,942)	135 (102–196)
**Unsuccessfully cannulated adrenal veins (*n* = 34)**		
Plasma aldosterone (pmol/L)	1105 (264–2133)	1005 (898–1648)
Serum cortisol (nmol/L)	720 (591–985)	677 (630–769)
Plasma normetanephrine (pmol/L)	370 (287–525)	347 (295–498)
Plasma metanephrine (pmol/L)	135 (85–304)	121 (95–175)

Concurrent cortisol autonomy was not identified in any of the patients. Cortisol physiology was formally assessed prior to the AVS in 30 out of 41 (73%) patients, through the use of urinary free cortisol measurement in 9 out of 36 (25%) patients, midnight salivary cortisol measurements in 1 out of 36 (3%) patients, and an overnight dexamethasone suppression test in 21 out of 36 (58%) patients. The investigating clinician decided not to investigate the possibility of concurrent cortisol autonomy (as outlined in the methods) in the remaining 11 (27%).

### Assessment of cannulation using metanephrine data

**Figure 1:**
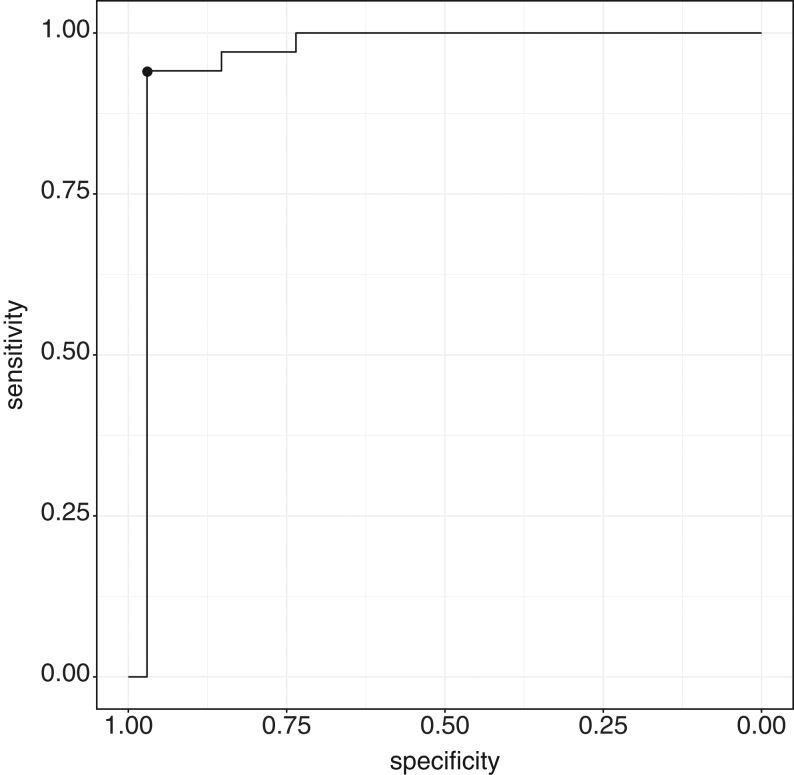
ROC curve for AVmet–PVmet ratio to assess successful cannulation based on the SIcort result. Optimum threshold: 26, sensitivity 94.1%, specificity 97.1%.

An AVmet–PVmet ratio of ≥12.0 to indicate successful cannulation showed strong agreement with the SIcort (*n* = 117, sensitivity 98%, specificity 89%, PPV 95%, NPV 94%) (Table 3). The agreement was entirely concordant when left adrenal vein data only was reviewed. The optimum metanephrine-derived SI was a ratio ≥21 (sensitivity 94% (95% CI 85–100), specificity 97% (95% CI, 91–100%)) (Fig. 1).

**Table 3:** Comparison of central-to-peripheral plasma metanephrine (AVmet–PVmet) ratio ≥12.0 to indicate successful cannulation with the SIcort.

*n* = 117		Successful cannulation (cortisol)		
		Yes	No	Total
AVmet–PVmet	≥12.0	80	4	84
	<12.0	2	31	33
	Total	82	35	117

Sensitivity 97.6%, Specificity 88.6%, PPV 95.2%, NPV93.9%

Results were discordant in six samples obtained from the attempted cannulation of the right AV. On two occasions, the use of the SIcort indicated successful AV cannulation, one with an SIcort of 5.75 but AVmet–PVmet of 10.5 and one with an SIcort of 14.10 but AVmet–PVmet of 2.8. Both patients were diagnosed with bilateral PA on the basis of the LIcort. On four occasions, the SIcort indicated unsuccessful AV cannulation (C-SI range 2.3–3.7), although it should be noted that the SIcort for one of these attempted cannulations was 3.7, greater than the threshold of ≥3.0 used by many centers. The AVmet–PVmet range was 12.2–19.1 in the three attempted cannulations where the SIcort was <3.0 but significantly higher at 556.7 where the SIcort was 3.7. On the basis of unsuccessful AVS, all four participants were managed medically. The participant where the AVmet–PVmet ratio supported cannulation had bilateral adrenal nodules on imaging. Use of a plasma metanephrine-derived lateralizing index (described later) supported bilateral PA (LImet 2.3).

### Assessment of cannulation using a plasma metanephrine–normetanephrine ratio

**Figure 2:**
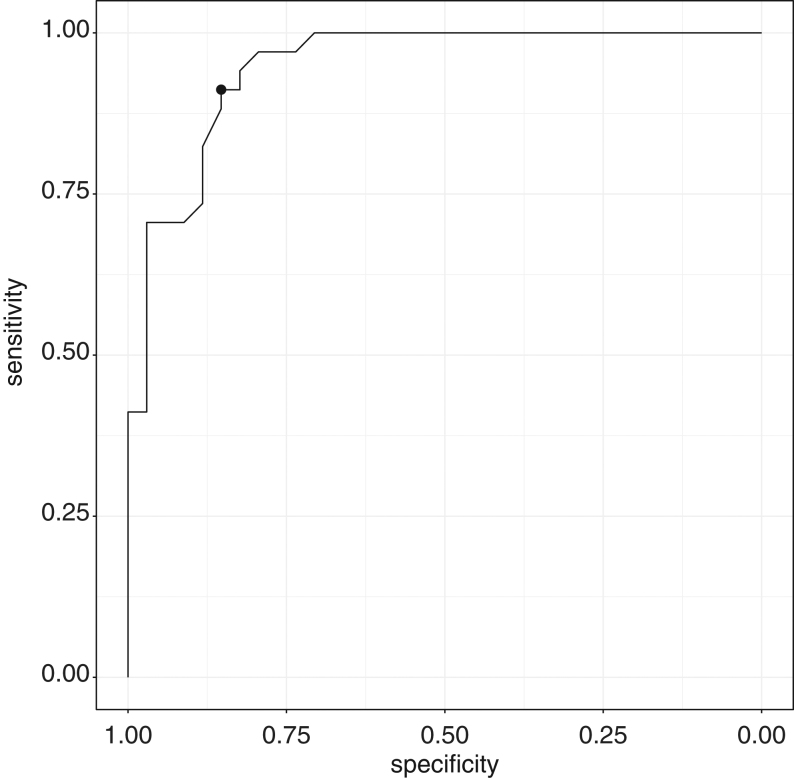
ROC curve for AVmet–AVnormet ratio to assess successful cannulation based on SIcort result. AUC 0.94, optimum threshold 1.91–2.0, sensitivity 91.2%, specificity 85.3%.

The mean adrenal vein–peripheral vein plasma metanephrine concentration in successfully cannulated adrenal veins (*n* = 83, *M*: 124.6) was significantly greater than corresponding concentrations for plasma normetanephrine (*n* = 37, *M*: 9.8). The optimum threshold ratio comparing use of an AVmet–AVnormet ratio against the standard SIcort to assess cannulation was 1.91–2.0 (sensitivity 91%, specificity 85%) (Fig. 2). An AVmet–AVnormet ratio ≥2.0 to indicate successful cannulation showed strong agreement with the SIcort of ≥5.0 (*n* = 117, sensitivity 93%, specificity 86%, PPV 94%, NPV 84%) (Table 4). In 11 attempted adrenal vein cannulations, the SIcort supported successful cannulation when the AVmet–AVnormet ratio did not on six occasions (range 0.77–1.97). The SIcort was in agreement with the AVmet–PVmet ratio in four of these participants and not in two. Use of the SIcort supported unsuccessful cannulation (range 2.1–3.7) in contrast to use of the AVmet–AVnormet ratio on five occasions (2.2–3.9). The participant reported above with a SIcort ≥3.0 (3.7) from one attempted AV cannulation and an Avmet–PVmet ratio supporting successful cannulation (566.7), had an AVmet–AVnormet ratio of 3.9, also suggesting successful cannulation.

**Table 4:** Comparison of central plasma metanephrine-to-plasma normetanephrine (AVmet–AVnormet) ratio of ≥2.0 to indicate successful cannulation with the SIcort.

*n* = 117		Successful cannulation (cortisol)		
		Yes	No	Total
AVmet–AVnormet	≥2.0	76	5	81
	<2.0	6	30	36
	Total	82	35	117

Sensitivity 92.7%, specificity 85.7%, PPV 93.8%, NPV 83.3%.

Use of this threshold to assess cannulation was 96% (112/117 cannulations) concordant with use of an AVmet–PVmet ratio of ≥12.0. The AVmet–AVnormet ratio suggested unsuccessful cannulation (range 1.62–1.84) in 4 successfully cannulated adrenal veins as determined by a cortisol SIcort and AVmet–PVmet ≥12.0 (range 57.7–353.7), and successful cannulation (2.66) in 1 unsuccessfully cannulated vein (AVmet–PVmet = 5.96).

### Assessment of lateralization using an aldosterone–metanephrine ratio

**Figure 3:**
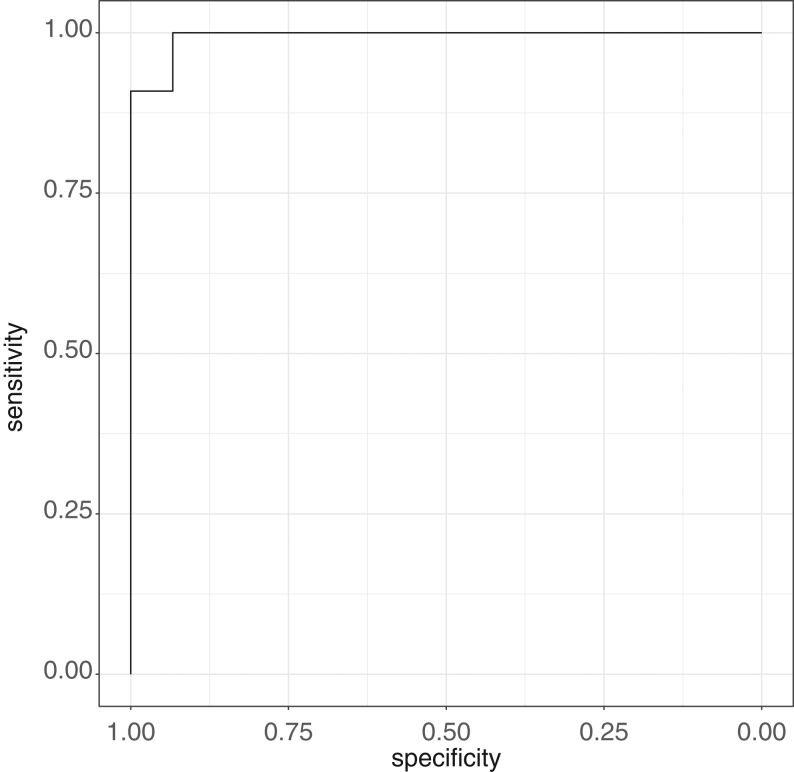
ROC curve for metanephrine lateralization index as compared to a reference cortisol lateralization index ≥ 4.0.

Twenty-six participants had successful cannulation of both AV by SIcort criteria, and had available plasma metanephrine data. A metanephrine-derived lateralizing index (LImet) of ≥4.0 showed strong agreement with the standard LIcort when assessing lateralization of aldosterone excess (*n* = 26, sensitivity 100%, specificity 94.1%, PPV 91.6%, NPV 100%) (Table 5, Fig. 3).

**Table 5:** Comparison of an LImet of ≥4.0 to the standard LIcort for assessing lateralization of aldosterone excess.

*n* = 26		LIcort		
		Lateralized	Non-lateralized	Total
LImet ratio	≥4.0	11	1	12
	<4.0	0	15	15
	Total	11	15	26

Sensitivity 100%, specificity 94.1%, PPV 91.6%, NPV 100%.

Only one participant had discordant results when comparing the LIcort and LImet, with the LIcort suggesting bilateral disease (3.49) and LImet lateralizing to the left adrenal gland (5.97). The SIcort, AVmet–PVmet, and AVmet–AVnormet ratios had all suggested successful cannulation bilaterally. CT imaging was reported as showing bilateral bulky adrenal glands, with the left larger than the right. On the basis of the LIcort, the patient was managed medically.

## Discussion

AVS remains a key component of the investigation of PA and enables the clinician to determine whether surgical resection of unilateral adrenal pathology is possible. This in turn presents a chance for cure, with improved blood pressure control and expected normalization of any potassium abnormality (33, 34, 35). Our study demonstrated that plasma metanephrines can be an adequate replacement for cortisol in the interpretation of AVS results based on the strong agreement between selectivity indices calculated with either one of these hormones. Our study is consistent with data on cannulation success in a similar-sized study (*n* = 51) reported by Dekkers and colleagues (28). In addition, our data suggest that use of an adrenal vein plasma metadrenaline-to-plasma normetadrenaline ratio to assess cannulation and an aldosterone-to-metanephrine ratio to assess lateralization compares favorably to the standard cortisol-derived indices.

The primary rationale to consider an AVS protocol that does not depend on the measurement of cortisol concentrations for both cannulation and lateralization is the possibility of abnormal cortisol physiology in those investigated for primary aldosteronism. A selectivity index based on cortisol measurements may lead to incorrect interpretation of the success of adrenal vein cannulation in patients with any degree of concurrent hyper- or hypocortisolemia, especially if caused by a unilateral cortisol-secreting adenoma. Exogenous glucocorticoid exposure is common (0.5–17% of various studied populations) and adrenal insufficiency is observed in 14% and 63% of this population (36, 37). Concurrent cortisol hypersecretion in patients with confirmed PA has been reported in multiple case reports and at a prevalence of up to 33% in case series (19, 20, 21, 22, 23). Recent studies have suggested the prevalence may be higher still when multiple assessments of cortisol physiology are performed (38, 39). Aldosterone/cortisol co-secreting adenomas tend to be larger than pure aldosterone-producing adenomas, and are associated with a significantly greater risk of diabetes and cardiovascular disease (20, 40, 41). Cortisol dysfunction is patients with PA has also been reported as a result of concurrent cortisol secreting adenomas and bilateral adrenal hyperplasia (42, 43, 44).

However, assessing for milder forms of cortisol dysfunction is not straightforward, and the optimal method of doing so is yet to be established (25, 26). While guidelines suggest using the 1mg overnight dexamethasone suppression test, there remains no consensus on the threshold that indicates mild cortisol autonomy, and there is a high frequency of false positives (25, 26, 45). ACTH can be measured to further validate results of a dexamethasone suppression test, but assay interference may impede interpretation (46). Neither salivary nor urinary cortisol testing is adequate to detect mild cortisol dysfunction (25). A single basal measurement of dehydroepiandrosterone sulfate (aka DHEA) has recently shown promise as a tool to detect mild autonomous cortisol secretion (47).

Acknowledging the above difficulties assessing for milder forms of cortisol autonomy, the possibility of abnormal cortisol physiology in PA indicates the need for alternatives to cortisol to assess selectivity and lateralization. Our data support the previously proposed AVmet–PVmet ratio of ≥12.0 to indicate successful AV cannulation (sensitivity 98%, specificity 89%) and suggested an optimal ratio of ≥21 (sensitivity 94%, specificity 97%).

As the conversion of norepinephrine to epinephrine is catalyzed by adrenal PNMT, we hypothesized that the increase in plasma metanephrine concentrations in the adrenal vein relative to the peripheral vein would be greater than the relative increase in plasma normetanephrine, within a successfully cannulated adrenal vein. This was evident in our data, with the mean adrenal vein plasma metanephrine concentration approximately 124 times the mean concentration of peripheral plasma metanephrine, while the mean adrenal vein plasma normetanephrine was just 10 times the mean concentration of peripheral plasma normetanephrine. Thus, we assessed whether use of an AVmet–Avnormet ratio to assess cannulation was comparable to outcomes obtained through other methods of assessing cannulation. Our data showed that an AVmet–AVnormet ratio ≥2.0 was 96% concordant with the use of a AVmet–PVmet ratio ≥12.0, and 93% concordant with the cortisol-derived SI. This observation requires assessment in further studies of AVS procedures in the context of both normal and abnormal cortisol physiology but may be of value if peripheral samples are for any reason not available to aid interpretation.

Lastly, our data showed that a metanephrine-derived lateralizing index (LImet) of ≥4.0 showed strong agreement with the standard LIcort with a specificity of 93% and a sensitivity of 100%. By contrast an LImet ≥3.0 (specificity 87%, sensitivity 100%) or ≥5.0 (specificity 93%, sensitivity 91%) were less concordant. In comparison, Buffalo and colleagues calculated an optimal LImet of 4.3 in their cohort, once patients with asymmetrical cortisol secretion had been excluded (30).

There are several limitations to our study to acknowledge. Our conclusions are limited by a small sample size, particularly the assessment of a lateralizing index incorporating aldosterone and plasma metanephrine measurements. This is partially attributable to higher than optimal failure rates for right adrenal vein cannulation, although this provided a larger volume of unsuccessful cannulation data with which to test the metanephrine-derived selectivity indices. A wide range of cortisol-derived SIs (1.1–5.0) are reported in the international literature, with an international panel suggesting a cortisol-derived SI of >3.0 as the cutoff for ACTH-stimulated AVS (17). In our center, we consider a stimulated SI of >5.0 to indicate successful cannulation; an analysis of our data using an SI of >3.0 revealed only one patient where this differing threshold would have Three from our cohort had indeterminate aldosterone measurements at the end of the saline infusion test and one had an aldosterone of <140 pmol/L, but investigations continued on the basis of clinical suspicion. Of these, the patient with an aldosterone of <140 pmol/L was diagnosed with right adrenal gland PA and underwent successful surgery, two were diagnosed with bilateral PA, and one had an unsuccessful AVS.

No patient in our study had concurrent hypercortisolemia based on testing performed, and 27% of patients in this cohort did not undergo formal testing of cortisol physiology. Acknowledging the difficulties assessing mild cortisol autonomy, as outlined previously, this is a limitation of our study. However, it remains unclear as to the impact that cortisol autonomy may have on AVS, and concern is primarily based on case reports and theoretical risk (48, 49). Low-grade cortisol autonomy did not substantially limit the interpretation of AVS data in a recent cohort of 144 individuals with PA, 15% of whom had evidence for cortisol autonomy, although these patients did have a lower SI on the side of unaffected adrenal gland as well as a lower LI in comparison to those with normal cortisol assessments (50). Further studies are required to assess the performance of metanephrine ratios in patients with concurrent cortisol dysfunction.

In conclusion, our data show strong agreement between a SIcort ≥5.0 and an AVmet–PVmet of ≥12.0 or AVmet–AVnormet of ≥2.0 to indicate successful cannulation, and the cortisol or metanephrine-derived LI >4 for determining lateralization. These findings may aid the interpretation of AVS results in people with abnormal cortisol physiology either as the result of concurrent cortisol autonomy or exogenous GC exposure. Further studies are required to assess the use of these thresholds in the context of confirmed concurrent cortisol autonomy and larger cohorts.

## Declaration of interest

The authors declare that there is no conflict of interest that could be perceived as prejudicing the impartiality of the study reported.

## Funding

This work did not receive any specific grant from any funding agency in the public, commercial, or not-for-profit sector.

## Data availability statement

The data generated during and analyzed during for this study are not publicly available but are available from the corresponding author on reasonable request.

## Author contribution statement

Jun Yang is a senior editor of *Endocrine Connections*. Jun Yang was not involved in the review or editorial process of this paper, on which she is listed as an author.

## References

[1] Funder JW Carey RM Mantero F Murad MH Reincke M Shibata H Stowasser M & Young WF. The management of primary aldosteronism: case detection, diagnosis, and treatment: an endocrine society clinical practice guideline. Journal of Clinical Endocrinology and Metabolism 2016 101 1889–1916. (10.1210/jc.2015-4061) 26934393

[2] Zennaro MC Boulkroun S & Fernandes-Rosa FL. Pathogenesis and treatment of primary aldosteronism. Nature Reviews. Endocrinology 2020 16 578–589. (10.1038/s41574-020-0382-4) 32724183

[3] Monticone S D'Ascenzo F Moretti C Williams TA Veglio F Gaita F & Mulatero P. Cardiovascular events and target organ damage in primary aldosteronism compared with essential hypertension: a systematic review and meta-analysis. Lancet Diabetes and Endocrinology 2018 6 41–50. (10.1016/S2213-8587(1730319-4) 29129575

[4] Monticone S Burrello J Tizzani D Bertello C Viola A Buffolo F Gabetti L Mengozzi G Williams TA Rabbia F, *et al.* Prevalence and clinical manifestations of primary aldosteronism encountered in primary care practice. Journal of the American College of Cardiology 2017 69 1811–1820. (10.1016/j.jacc.2017.01.052) 28385310

[5] Brown JM Siddiqui M Calhoun DA Carey RM Hopkins PN Williams GH & Vaidya A. The unrecognized prevalence of primary aldosteronism: a cross-sectional study. Annals of Internal Medicine 2020 173 10–20. (10.7326/M20-0065) 32449886 PMC7459427

[6] Alam S Kandasamy D Goyal A Vishnubhatla S Singh S Karthikeyan G & Khadgawat R. High prevalence and a long delay in the diagnosis of primary aldosteronism among patients with young‐onset hypertension. Clinical Endocrinology 2021 94 895–903. (10.1111/cen.14409) 33393127

[7] Parasiliti-Caprino M Lopez C Prencipe N Lucatello B Settani F Giraudo G Rossato D Mengozzi G Ghigo E Benso A, *et al.* Prevalence of primary aldosteronism and association with cardiovascular complications in patients with resistant and refractory hypertension. Journal of Hypertension 2020 38 1841–1848. (10.1097/HJH.0000000000002441) 32384388

[8] Käyser SC Dekkers T Groenewoud HJ, van der Wilt GJ Bakx JC van der Wel MC Hermus AR Lenders JW & Deinum J. Study heterogeneity and estimation of prevalence of primary aldosteronism: a systematic review and meta-regression analysis. Journal of Clinical Endocrinology and Metabolism 2016 101 2826–2835. (10.1210/jc.2016-1472) 27172433

[9] Muth A Ragnarsson O Johannsson G & Wängberg B. Systematic review of surgery and outcomes in patients with primary aldosteronism. British Journal of Surgery 2015 102 307–317. (10.1002/bjs.9744) 25605481

[10] Citton M Viel G Rossi GP Mantero F Nitti D & Iacobone M. Outcome of surgical treatment of primary aldosteronism. Langenbeck’s Archives of Surgery 2015 400 325–331. (10.1007/s00423-014-1269-4) 25567077

[11] Yang Y Reincke M & Williams TA. Prevalence, diagnosis and outcomes of treatment for primary aldosteronism. Best Practice and Research Clinical Endocrinology and Metabolism 2020 34 101365. (10.1016/j.beem.2019.101365) 31837980

[12] Turcu AF & Auchus R. Approach to the patient with primary aldosteronism: utility and limitations of adrenal vein sampling. Journal of Clinical Endocrinology and Metabolism 2021 106 1195–1208. (10.1210/clinem/dgaa952) 33382421 PMC7993592

[13] Jing Y Hu J Luo R Mao Y Luo Z Zhang M Yang J Song Y Feng Z Wang Z, *et al.* Prevalence and characteristics of adrenal tumors in an unselected screening population: a cross-sectional study. Annals of Internal Medicine 2022 175 1383–1391. (10.7326/M22-1619) 36095315

[14] Buffolo F Monticone S Williams TA Rossato D Burrello J Tetti M Veglio F & Mulatero P. Subtype diagnosis of primary aldosteronism: is adrenal vein sampling always necessary? International Journal of Molecular Sciences 2017 18 848. (10.3390/ijms18040848) 28420172 PMC5412432

[15] Gkaniatsa E Sakinis A Palmér M Muth A Trimpou P & Ragnarsson O. Adrenal venous sampling in young patients with primary aldosteronism. extravagance or irreplaceable? Journal of Clinical Endocrinology and Metabolism 2021 106 e2087–e2095. (10.1210/clinem/dgab047) 33507307

[16] Monticone S Viola A Rossato D Veglio F Reincke M Gomez-Sanchez C & Mulatero P. Adrenal vein sampling in primary aldosteronism: towards a standardised protocol. Lancet Diabetes and Endocrinology 2015 3 296–303. (10.1016/S2213-8587(1470069-5) 24831990

[17] Rossi GP Auchus RJ Brown M Lenders JWM Naruse M Plouin PF Satoh F & Young WF. An expert consensus statement on use of adrenal vein sampling for the subtyping of primary aldosteronism. Hypertension 2014 63 151–160. (10.1161/HYPERTENSIONAHA.113.02097) 24218436

[18] Dorin RI Qualls CR & Crapo LM. Diagnosis of adrenal insufficiency. Annals of Internal Medicine 2003 139 194–204. (10.7326/0003-4819-139-3-200308050-00009) 12899587

[19] Akehi Y Yanase T Motonaga R Umakoshi H Tsuiki M Takeda Y Yoneda T Kurihara I Itoh H Katabami T, *et al.* High prevalence of diabetes in patients with Primary Aldosteronism (PA) associated with subclinical hypercortisolism and prediabetes more prevalent in bilateral than unilateral PA: a large, multicenter cohort study in Japan. Diabetes Care 2019 42 938–945. (10.2337/dc18-1293) 31010944

[20] Späth M Korovkin S Antke C Anlauf M & Willenberg HS. Aldosterone- and cortisol-co-secreting adrenal tumors: the lost subtype of primary aldosteronism. European Journal of Endocrinology 2011 164 447–455. (10.1530/EJE-10-1070) 21270113

[21] Nakajima Y Yamada M Taguchi R Satoh T Hashimoto K Ozawa A Shibusawa N Okada S Monden T & Mori M. Cardiovascular complications of patients with aldosteronism associated with autonomous cortisol secretion. Journal of Clinical Endocrinology and Metabolism 2011 96 2512–2518. (10.1210/jc.2010-2743) 21593113

[22] Peng KY Liao HW Chan CK Lin WC Yang SY Tsai YC Huang KH Lin YH Chueh JS & Wu VC. Presence of subclinical hypercortisolism in clinical aldosterone-producing adenomas predicts lower clinical success. Hypertension 2020 76 1537–1544. (10.1161/HYPERTENSIONAHA.120.15328) 32921192

[23] Piaditis GP Kaltsas GA Androulakis II Gouli A Makras P Papadogias D Dimitriou K Ragkou D Markou A Vamvakidis K, *et al.* High prevalence of autonomous cortisol and aldosterone secretion from adrenal adenomas. Clinical Endocrinology 2009 71 772–778. (10.1111/j.1365-2265.2009.03551.x) 19226269

[24] Young WF. Diagnosis and treatment of primary aldosteronism: practical clinical perspectives. Journal of Internal Medicine 2019 285 126–148. (10.1111/joim.12831) 30255616

[25] Ueland GÅ Grinde T Methlie P Kelp O Løvås K & Husebye ES. Diagnostic testing of autonomous cortisol secretion in adrenal incidentalomas. Endocrine Connections 2020 9 963–970. (10.1530/EC-20-0419) 33032259 PMC7576642

[26] Araujo-Castro M Sampedro Núñez MA & Marazuela M. Autonomous cortisol secretion in adrenal incidentalomas. Endocrine 2019 64 1–13. (10.1007/s12020-019-01888-y) 30847651

[27] Eisenhofer G Kopin IJ & Goldstein DS. Catecholamine metabolism: a contemporary view with implications for physiology and medicine. Pharmacological Reviews 2004 56 331–349. (10.1124/pr.56.3.1) 15317907

[28] Dekkers T Deinum J Schultzekool LJ, Blondin D Vonend O Hermus AR Peitzsch M Rump LC Antoch G Sweep FC, *et al.* Plasma metanephrine for assessing the selectivity of adrenal venous sampling. Hypertension 2013 62 1152–1157. (10.1161/HYPERTENSIONAHA.113.01601) 24082051

[29] Goupil R Wolley M Ungerer J McWhinney B Mukai K Naruse M Gordon RD & Stowasser M. Use of plasma metanephrine to aid adrenal venous sampling in combined aldosterone and cortisol over-secretion. Endocrinology, Diabetes and Metabolism Case Reports 2015 2015 150075. (10.1530/EDM-15-0075) PMC4637894 26557366

[30] Buffolo F Pieroni J Ponzetto F, Forestiero V Rossato D Fonio P Nonnato A Settanni F Mulatero P Mengozzi G, *et al.* Prevalence of cortisol cosecretion in patients with primary aldosteronism: role of metanephrine in adrenal vein sampling. Journal of Clinical Endocrinology and Metabolism 2023 108 e720–e725. (10.1210/clinem/dgad179) 36974473

[31] Christou F Pivin E Maillard M Doenz F Pechère-Bertschi A Burnier M Grouzmann E & Wuerzner G. Comparison between cortisol, free metanephrines and the free/total metanephrines ratio to assess selectivity of adrenal vein sampling. Journal of Hypertension 2017 35(Supplement 2) e312. (10.1097/01.hjh.0000523921.15935.b4)

[32] Carroll RW Gould A Feltham J & Harper S. A case of confirmed primary hyperaldosteronism diagnosed despite normal screening investigations. New Zealand Medical Journal 2017 130 129–132. 28494487

[33] Vorselaars WMCM Nell S Postma EL Zarnegar R Drake FT Duh QY Talutis SD McAneny DB McManus C Lee JA, *et al.* Clinical outcomes after unilateral adrenalectomy for primary aldosteronism. JAMA Surgery 2019 154 e185842. (10.1001/jamasurg.2018.5842) 30810749 PMC6484800

[34] Vorselaars WMCM van Beek DJ Postma EL Spiering W Borel Rinkes IHM Valk GD Vriens MR & International CONNsortium Study Group. Clinical outcomes after surgery for primary aldosteronism: evaluation of the PASO-investigators’ consensus criteria within a worldwide cohort of patients. Surgery 2019 166 61–68. (10.1016/j.surg.2019.01.031) 31053245

[35] Sellgren F Koman A Nordenström E Hellman P Hennings J & Muth A. Outcomes after surgery for unilateral dominant primary aldosteronism in Sweden. World Journal of Surgery 2020 44 561–569. (10.1007/s00268-019-05265-8) 31720794

[36] Laugesen K Jørgensen JOL Petersen I & Sørensen HT. Fifteen-year nationwide trends in systemic glucocorticoid drug use in Denmark. European Journal of Endocrinology 2019 181 267–273. (10.1530/EJE-19-0305) 31269470

[37] Joseph RM Hunter AL Ray DW & Dixon WG. Systemic glucocorticoid therapy and adrenal insufficiency in adults: a systematic review. Seminars in Arthritis and Rheumatism 2016 46 133–141. (10.1016/j.semarthrit.2016.03.001) 27105755 PMC4987145

[38] Gerards J Heinrich DA Adolf C Meisinger C Rathmann W Sturm L Nirschl N Bidlingmaier M Beuschlein F Thorand B, *et al.* Impaired glucose metabolism in primary aldosteronism is associated with cortisol cosecretion. Journal of Clinical Endocrinology and Metabolism 2019 104 3192–3202. (10.1210/jc.2019-00299) 30865224

[39] Arlt W Lang K Sitch AJ Dietz AS Rhayem Y Bancos I Feuchtinger A Chortis V Gilligan LC Ludwig P, *et al.* Steroid metabolome analysis reveals prevalent glucocorticoid excess in primary aldosteronism. JCI Insight 2017 2. (10.1172/jci.insight.93136) PMC5396526 28422753

[40] Tang L Li X Wang B Ma X Li H Gao Y Gu L Nie W & Zhang X. Clinical characteristics of aldosterone-and cortisol-coproducing adrenal adenoma in primary aldosteronism. International Journal of Endocrinology 2018 2018 4920841. (10.1155/2018/4920841) 29770148 PMC5889857

[41] Yasuda S Hikima Y Kabeya Y Iida S Oikawa Y Isshiki M Inoue I Shimada A & Noda M. Clinical characterization of patients with primary aldosteronism plus subclinical Cushing’s syndrome. BMC Endocrine Disorders 2020 20 9. (10.1186/s12902-020-0490-0) 31931803 PMC6958814

[42] Ren K Wei J Liu Q Zhu Y Wu N Tang Y Li Q Zhang Q Yu Y An Z, *et al.* Hypercortisolism and primary aldosteronism caused by bilateral adrenocortical adenomas: a case report. BMC Endocrine Disorders 2019 19 63. (10.1186/s12902-019-0395-y) 31208392 PMC6580498

[43] Tokumoto M Onoda N Tauchi Y Kashiwagi S Noda S Toi N Kurajoh M Ohsawa M Yamazaki Y Sasano H, *et al.* A case of Adrenocoricotrophic hormone-independent bilateral adrenocortical macronodular hyperplasia concomitant with primary aldosteronism. BMC Surgery 2017 17 97. (10.1186/s12893-017-0293-z) 28877721 PMC5585945

[44] Teragawa H Oshita C Orita Y Hashimoto K Nakayama H Yamazaki Y & Sasano H. Primary aldosteronism due to bilateral micronodular hyperplasia and concomitant subclinical Cushing’s syndrome: a case report. World Journal of Clinical Cases 2021 9 1119–1126. (10.12998/wjcc.v9.i5.1119) 33644175 PMC7896658

[45] Fassnacht M Arlt W Bancos I Dralle H Newell-Price J Sahdev A Tabarin A Terzolo M Tsagarakis S & Dekkers OM. Management of adrenal incidentalomas: European society of endocrinology clinical practice guideline in collaboration with the European network for the study of adrenal tumors. European Journal of Endocrinology 2016 175 G1–G34. (10.1530/EJE-16-0467) 27390021

[46] Yener S Demir L Demirpence M Mahmut Baris M Simsir IY Ozisik S Comlekci A & Demir T. Interference in ACTH immunoassay negatively impacts the management of subclinical hypercortisolism. Endocrine 2017 56 308–316. (10.1007/s12020-017-1268-7) 28247312

[47] Liu MS Lou Y Chen H Wang YJ Zhang ZW Li P & Zhu DL. Performance of DHEAS as a screening test for autonomous cortisol secretion in adrenal incidentalomas: a prospective study. Journal of Clinical Endocrinology and Metabolism 2022 107 e1789–e1796. (10.1210/clinem/dgac072) 35137142

[48] Goupil R Wolley M Ahmed AH Gordon RD & Stowasser M. Does concomitant autonomous adrenal cortisol overproduction have the potential to confound the interpretation of adrenal venous sampling in primary aldosteronism? Clinical Endocrinology 2015 83 456–461. (10.1111/cen.12750) 25683582

[49] Reddy RPM & Brutsaert E. A case of adrenal adenoma co-secreting aldosterone and cortisol: limits of adrenal vein sampling. Journal of the Endocrine Society 2019 3(Supplement 1) SUN-372. (10.1210/js.2019-SUN-372)

[50] O'Toole SM Sze WC Chung TT Akker SA Druce MR Waterhouse M Pitkin S Dawnay A Sahdev A Matson M, *et al.* Low-grade cortisol cosecretion has limited impact on ACTH-stimulated AVS parameters in primary aldosteronism. Journal of Clinical Endocrinology and Metabolism 2020 105. (10.1210/clinem/dgaa519) 32785656

